# COVID-19 Vaccine: A Common Suspect but Rare Culprit in Drug Rash With Eosinophilia and Systemic Symptoms (DRESS) Syndrome

**DOI:** 10.7759/cureus.31310

**Published:** 2022-11-09

**Authors:** Mary Hanna, Samuel Yang

**Affiliations:** 1 Department of Family Medicine, Loma Linda University Health, Loma Linda, USA

**Keywords:** rash, eosinophilia, lymphadenopathy, antiseizure medication, dress syndrome

## Abstract

Drug rash with eosinophilia and systemic symptoms (DRESS) syndrome is a rare drug reaction that commonly presents with rash, fever, lymphadenopathy, eosinophilia, and multiorgan involvement. We present a case of this syndrome in a 31-year-old male who presented with a diffuse erythematous morbilliform rash with high fever and elevated liver enzymes. Upon history taking, the patient reported acute onset of multiple seizures that required intubation and ICU admission six weeks prior, which started 24 hours after receiving the Johnson and Johnson Janssen coronavirus disease 2019 (COVID-19) vaccine. During that hospitalization, he was given antiseizure medications Keppra (levetiracetam) and Dilantin (phenytoin), which he was eventually discharged home with. During our encounter with the patient, Dermatology was consulted and recommended punch skin biopsy, which revealed spongiotic dermatitis with subcorneal pustules along with superficial perivascular and mixed lymphocytic and neutrophilic infiltrate with dermal edema and rare eosinophils. Given these findings in conjunction with the patient’s fever, elevated liver function, and cervical lymphadenopathy, the rash was consistent with DRESS syndrome or a pustular drug eruption likely secondary to phenytoin or levetiracetam. This case was eventually resolved with treatment with oral and topical corticosteroids and close outpatient follow-up with Dermatology. Prompt diagnosis and treatment of DRESS syndrome are therefore critical as the mortality rate can be as high as 10% in the setting of liver failure.

## Introduction

Drug reaction with eosinophilia and systemic symptoms (DRESS) is a rare, but life-threatening condition that commonly presents with a diffuse rash, fever, lymphadenopathy, eosinophilia, and multiorgan involvement commonly involving the liver after drug exposure [1]. It is a delayed type IV hypersensitivity drug reaction. Generally, symptoms of DRESS will begin about two to eight weeks after the initiation of the offending drug [1]. Its etiology has been linked with lymphocyte activation, drug metabolic enzyme defects, eosinophilia, and human herpesvirus-6 reactivation [2]. The most common drugs to cause DRESS syndrome are antiseizure medications, antibiotics, and allopurinol [1]. The incidence of DRESS associated with antiepileptic drugs is reported at one in 1000 to one in 10,000 exposures with a mortality rate of about 10%, most commonly from fulminant hepatitis with hepatic necrosis [2-4].

## Case presentation

We present a case of a 31-year-old male, adopted as a 10-day-old infant, with a history of intrauterine drug exposure and otherwise normal development and health. The patient presented to the urgent care with a diffuse erythematous morbilliform intense pruritic rash, and high fever (103F) for five days prior to presentation and about six weeks after receiving his coronavirus disease 2019 (COVID-19) vaccine.

The patient was hospitalized six weeks earlier to his current presentation for reported acute onset of multiple seizures that required intubation and ICU admission, which started 24 hours after receiving the Johnson and Johnson Janssen COVID-19 vaccine. He was discharged on prophylactic Keppra (levetiracetam) and Dilantin (phenytoin). One month after being on the antiepileptic drugs, he was re-admitted for worsening seizures. Workup during that hospitalization revealed psychogenic seizures and Keppra and Dilantin were both discontinued. Then two weeks later, he presented with the symptoms of rash and high fever, which the patient believed may have been related to the COVID-19 vaccine.

The patient reported that the rash initially started five days prior to presentation as a small rash on his right upper extremity and eventually spread throughout his entire body. Three days after the onset of the rash, he was evaluated in the urgent care setting where he was prescribed oral methylprednisolone and hydroxyzine and discharged home. He reported that since taking the medications, his rash had improved in terms of redness, swelling, and itchiness but continued to spread throughout his body.

The patient denied any new medication use in the last one to two months other than Dilantin and Keppra. He reported occasional non-steroidal anti-inflammatory drugs (NSAIDs) and Tylenol use. He also denied using any other over-the-counter medications as well as other significant past medical history. He denied shortness of breath, dyspnea, chest pain, abdominal pain, nausea, vomiting, diarrhea, constipation, dizziness, or headaches.

**Figure 1:**
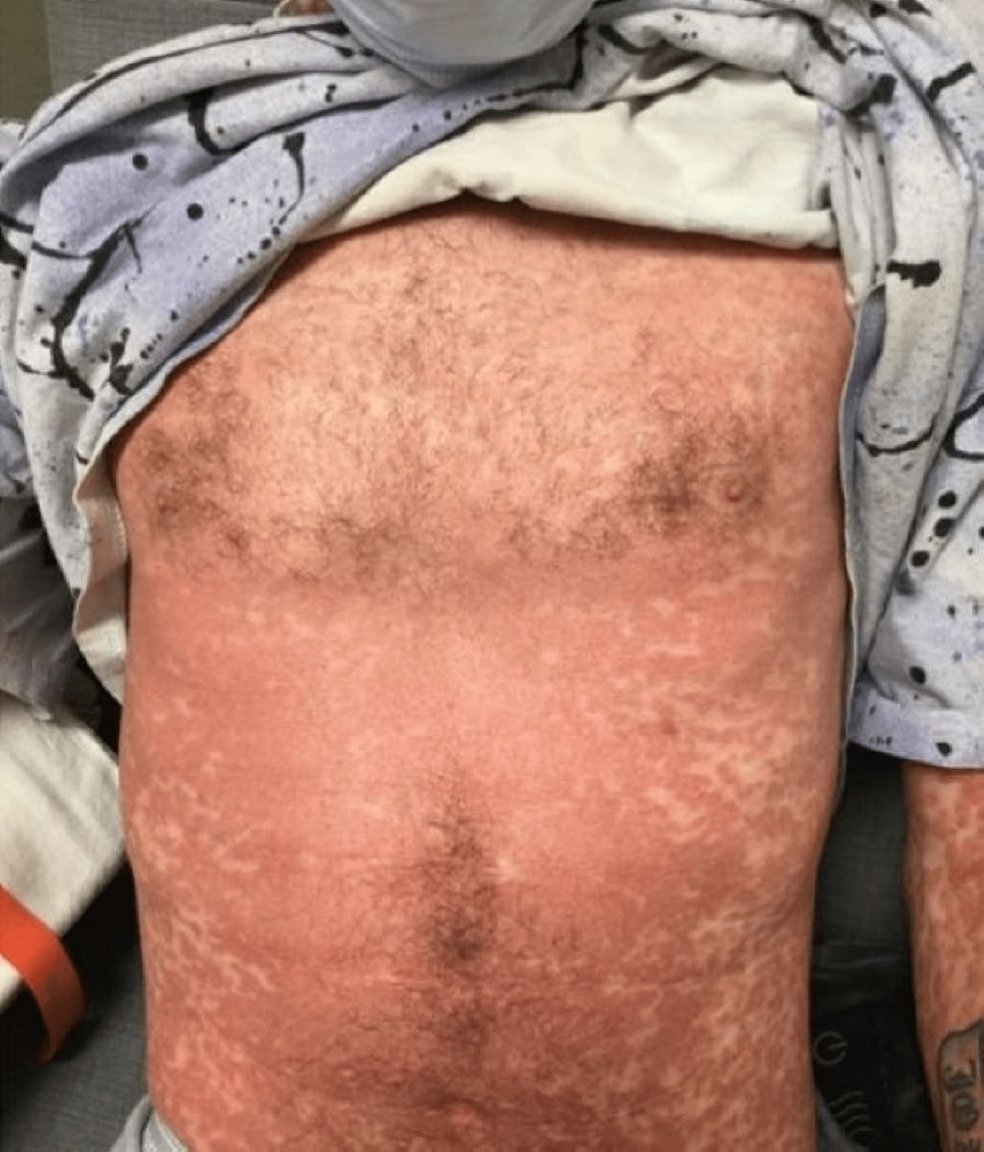
Rash at the time of presentation

**Figure 2:**
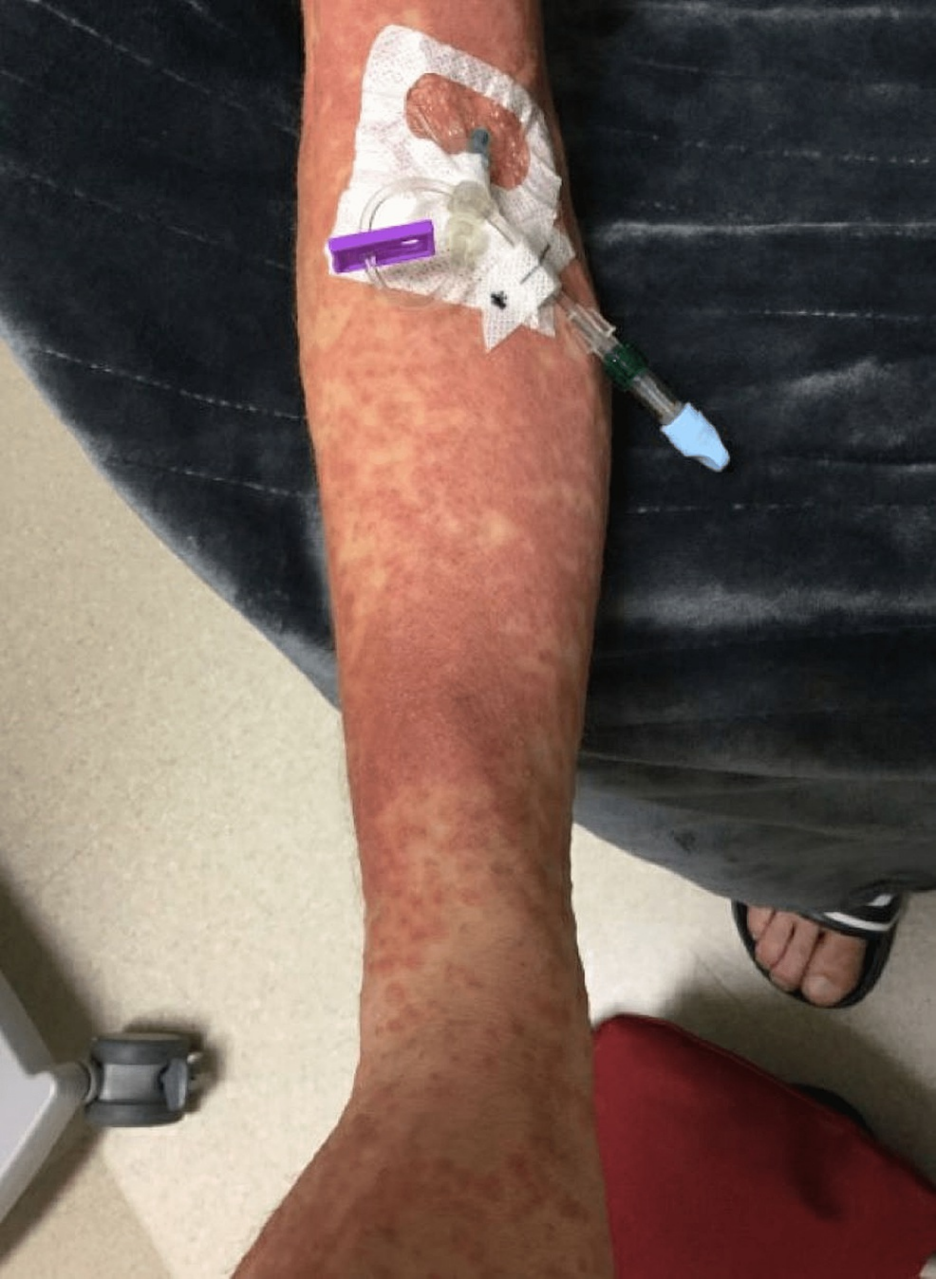
Rash at the time of presentation

**Figure 3:**
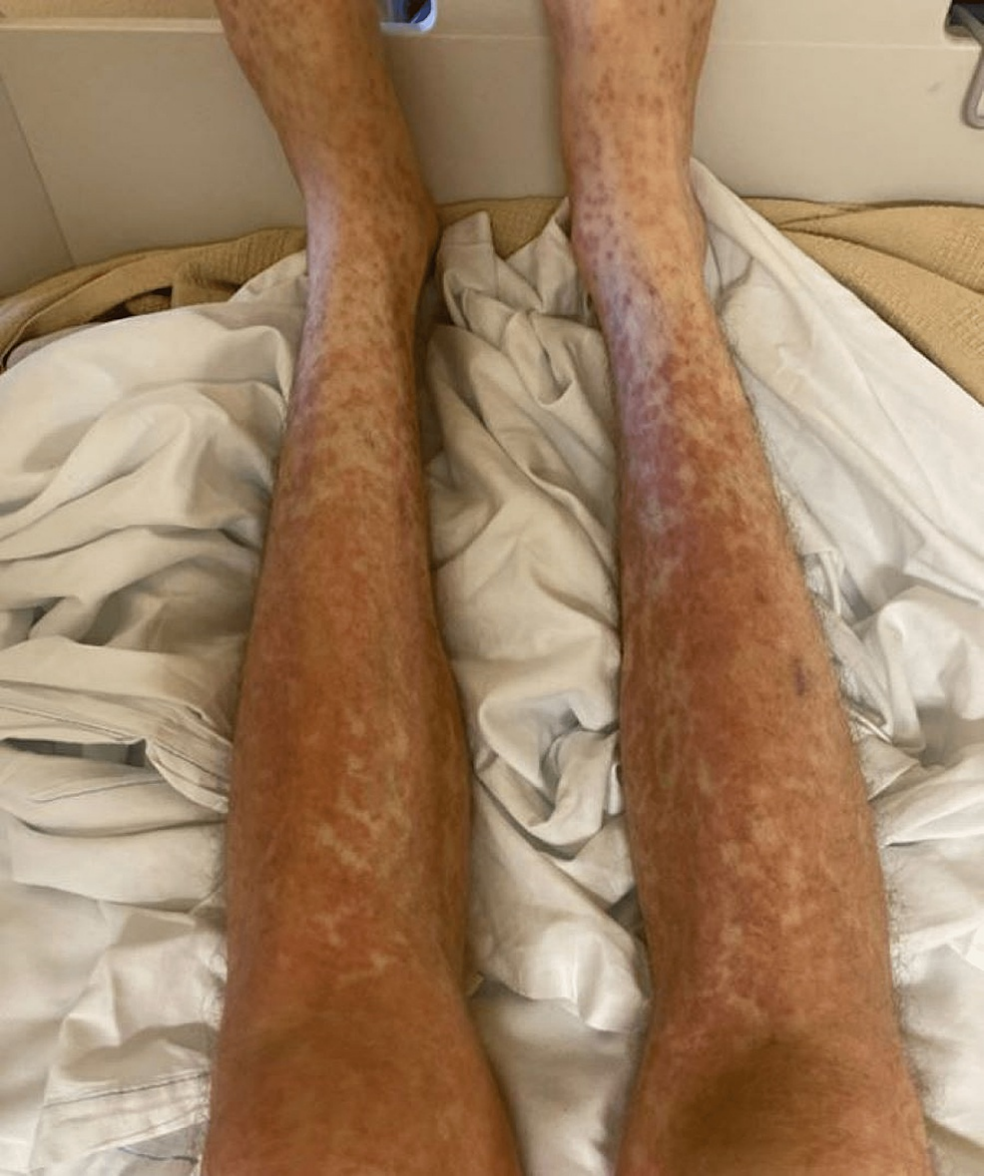
Rash at the time of presentation

Initial vitals on admission: blood pressure (BP) 125/74, heart rate (HR) 91/minute, temperature 99.1 F (37.3 °C), respiration rate 20/minute, and saturation of peripheral oxygen (SpO2) 96% on room air. The following day after admission, the patient had multiple fevers with a maximum temperature (Tmax) of 103 °F. Skin examination of the scalp, face, lips, neck, chest, axilla, back, abdomen, left arm, right arm, digits, nails, left leg and right leg were performed. All areas were normal except for morbilliform eruption with confluent erythema on the patient’s chest, abdomen, back, penis, scrotum, and upper and lower extremities and approaching erythroderma with pustule formation and palpable purpura on lower extremities. No mucosal or oral lesions were noted. The patient was also noted to have bilateral inguinal, right cervical, and right axillary lymphadenopathy. The patient otherwise had normal examination findings of his heart, lung, and abdomen (Figures 1, 2, 3).

Initial labs showed an elevated total white blood cell count of 19.57 (reference range 4.8-11.8 bil/L), hemoglobin at 13.2 (reference range 11.0-16.0 g/dL), and platelet at 293 (reference range 140-340 bil/L). A complete metabolic panel (CMP) revealed transaminitis with aspartate aminotransferase (AST) at 310 (reference range 0-35 U/L) and alanine transaminase (ALT) at 996 (reference range 7-45 U/L). C-reactive protein (CRP) was 6.0 (reference range 0.0-0.8 mg/dL) and erythrocyte sedimentation rate (ESR) Westergren was 31 (reference range 0-9 mm/hr). Additional labs showed an absolute eosinophil count of 1000 cells per microliter of blood. His antinuclear antibody (ANA) panel, acute hepatitis panel, syphilis, and HIV tests were all negative. Abdominal ultrasound revealed hepatomegaly with a 1.6 cm hyperechoic right lobe lesion, which is favored to represent hemangioma.

A skin punch biopsy was performed on the patient’s right upper back and right upper thigh. The biopsy revealed spongiotic dermatitis with subcorneal pustules along with superficial perivascular and mixed lymphocytic and neutrophilic infiltrate with dermal edema and rare eosinophils. There was no active vasculitis or atypical lymphoid infiltrate seen.

Dermatology recommended, in addition to triamcinolone ointment, starting prednisone at 1 mg/kg/day (60 mg daily) and monitoring daily complete blood count (CBC), CMP, and prothrombin time/partial thromboplastin time (PT/PTT) while hospitalized. Upon starting oral and topical steroids, the patient’s rash and liver enzymes significantly improved. The patient was discharged four days after initiation of treatment on the same dose of prednisone with a plan for a close follow-up with dermatology.

In the outpatient setting, the patient’s rash continued to improve. The patient followed up with dermatology about 1 week after his hospital discharge with a plan to continue long-term prednisone 40 mg daily for one month with steroid taper afterward. He was also recommended to continue triamcinolone 0.1% ointment as well. Eventually, his rash resolved completely, and liver enzymes normalized with steroid taper over eight weeks. (Table 1)

**Table 1:** Timeline of patient presentation ICU: Intensive Care Unit, EEG: Electroencephalogram, SIRS: Systemic Inflammatory Response Syndrome, CBC: Complete Blood Count, CMP: Comprehensive Metabolic Panel, COVID-19: Coronavirus Disease 2019

Date	Events	Diagnostic test and intervention
Day 0	Received Johnson & Johnson COVID-19 vaccination	
Day 1	Acute onset of seizures, admitted to the ICU	Antiseizure medication (Dilantin and Keppra) started
Day 31	Admitted to our hospital for seizures	Video-assisted EEG during his three-day hospitalization, Dilantin and Keppra medicines were discontinued
Day 36	Rash started	
Day 39	Urgent care visit	The patient was given oral methylprednisolone and hydroxyzine with minimal relief.
Day 41	Admission to our hospital for SIRS, rash, and transaminitis	CBC with differential (eosinophilia and transaminitis) confirmed with lab that eosinophilic absolute count was 1000, Skin biopsy, Dermatology consult: start prednisone 60 mg daily, monitor daily CBC and CMP
Day 45	Significant resolution of rash and down trending of liver enzymes Discharge from the hospital	Topical and oral steroids continued Dermatology follow-up as outpatient; Neurology follow-up as outpatient
Day 51	Dermatology visit; rash continued to improve but still present; liver enzymes continued to trend down	Continue prednisone 40 mg for one month, then plant to taper in smaller increments as tolerated; continue triamcinolone 0.1% ointment twice daily to body rash and can mix 50/50 with -Vaseline to face;. repeat labs in one month

## Discussion

DRESS syndrome can often be a tricky clinical diagnosis due to the variable presentation of the syndrome, its delayed onset, and its prolonged course even after stopping the culprit drug. Given the patient’s fever, elevated liver function, eosinophilia, and cervical lymphadenopathy (LAD), the rash was consistent with a drug rash that, in the full clinical context, was consistent with DRESS syndrome or a pustular drug eruption likely secondary to phenytoin or levetiracetam as those were the only new medications recently started.

While phenytoin is traditionally a more common culprit, levetiracetam can also cause DRESS syndrome, although much more rarely implicated [3,5]. More than 50 drugs have been reported to be associated with DRESS syndrome [6,7]. In patients who have recently started on new medications, clinicians need to be aware of this potentially fatal syndrome. The term drug-induced hypersensitivity syndrome (DIHS) is sometimes used alternatively or in addition to DRESS [2].

The clinical presentation during the acute phase of the disease classically presents with cutaneous eruption, fever, lymphadenopathy, hematologic abnormalities, and visceral organ involvement. Skin involvement is the most common presentation in form of symmetrical maculopapular (morbilliform) eruption. Mucosal involvement can occur in DRESS in the mild form [2,8]. The most common sign is fever ≥ 38.5 °C, followed by lymphadenopathy. Hematologic abnormalities typically include eosinophilia though atypical lymphocytosis, leukocytosis, neutrophilia, and monocytosis are observed in more than half of patients with DRESS syndrome [2,3,8]. While DRESS syndrome can affect other visceral organs, the liver is the most involved [2,3,6,8].

The exact pathogenesis is not well understood; however, a few mechanisms are thought to predispose individuals to DRESS syndrome, including: (1) A genetic deficiency in detoxification enzymes, therefore causing accumulation of drug metabolites, which results in an immunological response. This is thought to be a reason why DRESS syndrome is seen more among slow acetylators; (2) A genetic association of DRESS syndrome and certain human leucocyte antigen (HLA), which suggests another genetic role in the pathogenesis of DRESS syndrome; (3) A possible viral reactivation, particularly of human herpes virus-6 (HHV6), Epstein-Barr virus (EBV), and cytomegalovirus (CMV), induced by certain drugs leading to viral infection-induced drug reactions [6,8-10]. In terms of mortality, it is known that liver damage caused by lymphocyte infiltration is the main cause of mortality [6,9,10].

DRESS diagnosis can be made using any of three different sets of criteria: European registry of severe cutaneous adverse reactions to drugs and collection of biological samples (RegiSCAR) criteria, Bocquet’s criteria, and the Japanese consensus group to diagnose DIHS/DRESS syndrome. RegiSCAR criteria comprise at least three of the following seven characteristics: (1) fever >38 °C; (2) skin eruption; (3) lymphadenopathy involving at least two sites; (4) involvement of at least one internal organ; (5) lymphocytosis (> 4000/mm3) or lymphocytopenia (< 1500/mm3); (6) blood eosinophilia (> %10 or 700/mm3); and (7) thrombocytopenia (< 120.000/mm3 ) [6,10]. The Japanese DIHS criteria require evidence for the reactivation of HHV-6 for diagnosis, which is not included in RegiSCAR or Bocquet's criteria [10,11].

The recognition of the syndrome and immediate cessation of the culprit drug are the most important steps for appropriate management. Topical steroids can be used to treat the rash; however, systemic steroids are often needed, which are usually tapered over weeks or months to protect the organs from damage. Labs should be monitored carefully during this time. Experts recommend oral prednisolone 40 to 60 mg daily during active disease, while others recommend methylprednisolone I.V. at 1 mg/kg or (1.5 mg/kg) depending on disease severity and comorbidities. IV Methylprednisolone is preferred in DRESS patients to bypass potential decreased absorption in hospitalized patients and to avoid liver metabolism of prednisone into its active form [6,12].

Other treatment alternatives can be considered in patients where systemic steroids are contraindicated. Cyclosporine has been successfully used, given its rapid onset of action as an inhibitor of T-cell proliferation [11]. The French Society of Dermatology recommends intravenous immunoglobulin (IVIG) (2 gr/kg) for five days, especially in patients with life-threatening internal organ involvement. Gancyclovir has been suggested in cases with confirmation of viral reactivation of HHV [6,8,12].

The average time to recovery is six to nine weeks and most patients do well. Routine follow-up is recommended for about two years since some patients can suffer long-term organ dysfunction or develop autoimmune diseases [11,12]

Although adverse events from vaccines are rare, there are a few case reports of DRESS syndrome that could have been potentially triggered by vaccines including the COVID-19 vaccine [13-15]. In the setting of a recent viral pandemic and strong anti-science and anti-vaccine sentiment, the COVID-19 vaccine has become a convenient target for a variety of side effects, symptoms, and diseases to attribute to. In our case, the COVID-19 vaccine was initially considered a culprit for the patient’s presentation, which included seizures, rash, and high fever. However, after a thorough investigation, it was deemed more likely the vaccine itself was likely a bystander as the patient had been exposed to more known classically implicated drugs in Keppra and Dilantin. Thus, a detailed history, complete case review, lab evaluation, and a broad differential are needed to determine the culprit in patients presenting with a rash.

## Conclusions

DRESS syndrome is a rare condition commonly associated with certain drugs including anticonvulsants, antibiotics, and allopurinol. The primary presentations are rash, fever, lymphadenopathy, eosinophilia, and one or more organ injury involvement. The inclusion criteria and scoring system can be used for diagnosis with RegiSCAR being the most used. Treatment is mainly stopping the culprit drug and starting systemic steroids based on the severity of the disease. Prompt diagnosis and treatment of DRESS syndrome are critical due to its high morbidity and mortality. Close monitoring and follow-up are indicated to ensure avoiding further future complications. 

## References

[1] Shrestha R, Jha SK, Bartaula J (2021). Drug reaction with eosinophilia and systemic symptom (DRESS) following rifampicin treatment: a case report. Cureus.

[2] Husain Z, Reddy BY, Schwartz RA (2013). DRESS syndrome: Part I. Clinical perspectives. J Am Acad Dermatol.

[3] Cardoso CS, Vieira AM, Oliveira AP (2011). DRESS syndrome: a case report and literature review. BMJ Case Rep.

[4] Chen YC, Chiu HC, Chu CY (2010). Drug reaction with eosinophilia and systemic symptoms: a retrospective study of 60 cases. Arch Dermatol.

[5] Dar WR, Sofi N, Latief M, Dar IA, Kasana BA (2016). Levetiracetam induced drug reaction with eosinophilia and systemic symptom syndrome. Indian J Dermatol.

[6] Karakayalı B, Yazar AS, Çakir D (2017). Drug reaction with eosinophilia and systemic symptoms (DRESS) syndrome associated with cefotaxime and clindamycin use in a 6-year-old boy: a case report. Pan Afr Med J.

[7] Bluestein SB, Yu R, Stone C Jr, Phillips EJ (2021). Reporting of drug reaction with eosinophilia and systemic symptoms from 2002 to 2019 in the US Food and Drug Administration adverse event reporting system. J Allergy Clin Immunol Pract.

[8] Mukit W, Cooper R, Moudgil H, Ahmad N (2020). DRESS syndrome: an important differential for eosinophilia with systemic organ dysfunction. BMJ Case Rep.

[9] Descamps V, Ranger-Rogez S (2014). DRESS syndrome. Joint Bone Spine.

[10] Kim DH, Koh YI (2014). Comparison of diagnostic criteria and determination of prognostic factors for drug reaction with eosinophilia and systemic symptoms syndrome. Allergy Asthma Immunol Res.

[11] Shiohara T, Kano Y, Hirahara K, Aoyama Y (2017). Prediction and management of drug reaction with eosinophilia and systemic symptoms (DRESS). Expert Opin Drug Metab Toxicol.

[12] Schunkert EM, Divito SJ (2021). Updates and insights in the diagnosis and management of DRESS syndrome. Curr Dermatol Rep.

[13] Korekawa A, Nakajima K, Fukushi K, Nakano H, Sawamura D (2022). Three cases of drug-induced hypersensitivity syndrome associated with mRNA-based coronavirus disease 2019 vaccines. J Dermatol.

[14] O'Connor T, O'Callaghan-Maher M, Ryan P, Gibson G (2022). Drug reaction with eosinophilia and systemic symptoms syndrome following vaccination with the AstraZeneca COVID-19 vaccine. JAAD Case Rep.

[15] Lospinoso K, Nichols CS, Malachowski SJ, Mochel MC, Nutan F (2021). A case of severe cutaneous adverse reaction following administration of the Janssen Ad26.COV2.S COVID-19 vaccine. JAAD Case Rep.

